# A Comparative Analysis of Medial Versus Lateral Unicompartmental Arthroplasty: A Case-Matched Analysis of Patient-Reported Outcomes

**DOI:** 10.7759/cureus.98308

**Published:** 2025-12-02

**Authors:** Rohan Bassi, Kamparsh Thakur, Varun Vig, Divya Prakash

**Affiliations:** 1 Trauma and Orthopedics, Sandwell and West Birmingham NHS Trust, Birmingham, GBR

**Keywords:** case-matched, forgotten joint score, knee arthroplasty, lateral unicompartmental, medial unicompartmental

## Abstract

Introduction

Unicompartmental knee arthroplasty (UKA) is a bone-preserving alternative to total knee arthroplasty (TKA) for isolated compartmental osteoarthritis (OA). The medial compartment is most frequently affected due to varus malalignment, while lateral disease, associated with valgus alignment, is less common. Although UKA offers faster recovery and improved function compared to total knee replacement, direct comparisons between medial and lateral UKA outcomes are limited. This study aims to compare patient-reported outcomes between medial and lateral UKA using a retrospective case-matched design.

Methods

A retrospective case-matched study was conducted on patients who underwent fixed-bearing UKA performed by a single fellowship-trained surgeon between 2008 and 2023. All procedures followed a standardized surgical and rehabilitation protocol. Lateral UKA cases were matched 1:1 with medial UKA cases for age (±1 year), sex, and follow-up duration (±6 months). The primary outcome was the Forgotten Joint Score-12 (FJS-12) at the latest follow-up. Statistical comparisons used independent t-tests for continuous variables and chi-squared or Fisher’s exact tests for categorical data, with significance set at p < 0.05. Effect sizes were quantified using Cohen’s d.

Results

The cohort included equal numbers of medial (n = 25) and lateral (n = 25) UKA cases with no significant differences in demographics. The mean age was 59.3 years, and the mean follow-up was 7.6 years. The mean normalized FJS-12 scores were 56.8 (standard deviation {SD}: 37.7) for medial UKA and 57.4 (SD: 36.0) for lateral UKA (p = 0.86), with a negligible effect size (Cohen’s d = 0.05). The item-by-item analysis of the 12 components of the FJS-12 revealed no statistically significant differences between groups across activity-specific domains, including walking, standing, climbing stairs, and sports participation. The graphical distribution of FJS-12 scores showed substantial overlap between the two cohorts. No implant revisions or major complications were recorded in either group.

Conclusion

In this case-matched analysis of medial and lateral UKA performed by a single surgeon, patient-reported outcomes were equivalent at mid-term follow-up. Both medial and lateral UKA demonstrated comparable joint “forgettability” and functional integration, with no revisions observed. These findings indicate that, when performed with appropriate patient selection and consistent surgical technique, lateral UKA can achieve outcomes equivalent to medial UKA. The results support the safe and effective use of lateral UKA as a viable option for isolated lateral compartment disease.

## Introduction

Unicompartmental knee osteoarthritis (OA) is a common manifestation of degenerative knee disease, often resulting from altered limb alignment and chronic biomechanical stress. Varus malalignment predominantly affects the medial compartment, whereas valgus alignment places greater stress laterally. Additional factors such as meniscal deficiency, trauma, and ligamentous instability also contribute to compartment-specific degeneration [1]. The medial compartment remains the most frequently involved site, where cartilage loss typically initiates anteriorly and progresses posteriorly, often with the preservation of the anterior cruciate ligament (ACL) [2]. In contrast, lateral compartment arthritis is less predictable in progression. Published studies have shown that valgus malalignment increases the odds of lateral compartment OA progression by fourfold over an 18-month observation period [3]. For isolated compartmental disease refractory to nonoperative treatment, unicompartmental knee arthroplasty (UKA) offers a bone-preserving surgical alternative with faster recovery and lower morbidity compared to total knee arthroplasty (TKA). Fixed-bearing and mobile-bearing UKA designs are both widely used. Fixed-bearing implants offer surgical simplicity and lower dislocation risk, while mobile-bearing designs better replicate native knee kinematics by allowing axial rotation [4].

UKA survival rates have improved with modern implants and refined patient selection. A recent systematic review of over 30000 UKA procedures reported an overall revision rate of 8.8% at a mean follow-up of 6.5 years [5]. In medial UKA, common failure modes include aseptic loosening, bearing dislocation, and progressive lateral compartment disease [6]. Despite lower clinical thresholds for revisions in UKA compared with TKA, UKA has also been associated with better patient-reported outcomes, shorter hospital stays, and faster functional recovery [7,8]. Lateral UKA, though less commonly performed, has shown encouraging results in selected patients. A review of 25 years of registry data showed a five-year revision rate of 7.3% for lateral UKA [9]. Moreover, annual revision rates appear comparable between medial and lateral UKA, and short-term functional outcomes such as the Oxford Knee Score (OKS) have not demonstrated significant differences between the two [10]. Despite these encouraging results, direct comparative studies between medial and lateral UKA are limited, especially those using case-matched methodology. The lower frequency of lateral UKA limits the statistical power of many published series. This study aims to address this gap by performing a retrospective case-matched analysis comparing patient-reported outcomes between medial and lateral UKA performed by a single surgeon over a two-decade period.

## Materials and methods

Study design

This was a retrospective case-matched study comparing patient-reported outcomes of medial versus lateral unicompartmental knee arthroplasty (UKA) performed by a single fellowship-trained arthroplasty surgeon between 2008 and 2023. The study was registered with the Trust Audit Module Database (via SafeGuard Webportal by Ulysses) by the ID number 3122. All procedures were performed using a standardized medial or lateral parapatellar approach under regional or general anesthesia. Soft tissue dissection was done as considered appropriate. Fixed-bearing implants were used for all cases. All patients underwent the same perioperative protocols, including antibiotic prophylaxis, thromboembolism prevention, and accelerated rehabilitation. The primary outcome at the latest follow-up was the Forgotten Joint Score-12 (FJS-12), a validated patient-reported outcome measure (PROM) [11]. Complications, failure modes, and revision causes were recorded.

Inclusion and exclusion criteria

The inclusion criteria incorporated all adults who underwent primary UKA for any indication and in any sector. Body mass index (BMI) and comorbidities were not taken into consideration. Those with predominant osteoarthritis in the lateral compartment were categorized as lateral UKA, as it is extremely rare to find other compartments unaffected in these cases. Exclusion criteria included patients who had inflammatory arthropathy and patients who previously had surgery on the same knee.

Data collection

Patients were identified from a prospectively maintained surgical database, the surgeon’s National Joint Registry (NJR) data and the surgeon’s prospectively maintained database. All available lateral UKA cases were included as the basis for group-matching, as lateral UKA operative volume is known to be low in practice. No lateral UKA cases had been revised since; this assisted with case-matching. Medial UKA patients were matched in a 1:1 ratio with lateral UKA patients for age (±1 year), sex, and follow-up duration (±6 months). Each patient was contacted via telephone by a clinician who had not previously been involved in any of the arthroplasty cases. The original operating team was not involved in the data collection. This minimized any potential interviewer bias in the study. The 12 responses of the FJS questionnaire were recorded. All questions were asked in a consistent manner in English and related specifically to the artificial joint of interest.

Statistical analysis

Descriptive statistics were generated for demographical data, which included the mean and standard deviation (SD) for age and follow-up duration. Proportions were expressed for gender. To assess the comparison of the two cohorts, independent t-tests were performed for continuous data. Categorical data were compared with chi-squared (or Fisher’s exact test where appropriate), and significance was set at p < 0.05.

The FJS-12 was summated as a raw total score for each patient initially, which was then converted to a normalized total score (out of 100). The normalized total FJS was compared between the two cohorts using an independent t-test. The effect size was quantified using Cohen’s d value. For the individual scores of the FJS components, the calculation of the independent t-test result and an effect size using Cohen’s d value was performed. Statistical significance was set at p < 0.05. The distribution of FJS-12 scores and the comparison of total normalized scores were illustrated using histograms and boxplots, as appropriate.

## Results

The study cohort comprised equal numbers of medial and lateral unicompartmental knee arthroplasty (UKA) cases. Descriptive statistical data of the two groups are shown in Table 1. Using the independent t-test and chi-squared test for continuous and categorical data, respectively, it was shown that there was no statistically significant difference between the two cohorts in terms of gender, age at procedure, and follow-up duration (Table 1).

**Table 1:** Descriptive data analysis with statistical tests including the chi-squared test and independent t-test, as appropriate, generating p-values. UKA, unicompartmental knee arthroplasty; SD, standard deviation

Group variable	Medial UKA	Lateral UKA	Test statistic value (chi-squared value or t-test)	P-value
Male, n	10	9	0.035	0.850
Female, n	15	16		
Mean age at procedure (years) ± SD	58.61 ± 9.67	60.01 ± 11.48	0.241	0.810
Mean follow-up duration (years) ± SD	7.62 ± 3.72	7.65 ± 4.45	0.197	0.845

**Figure 1:**
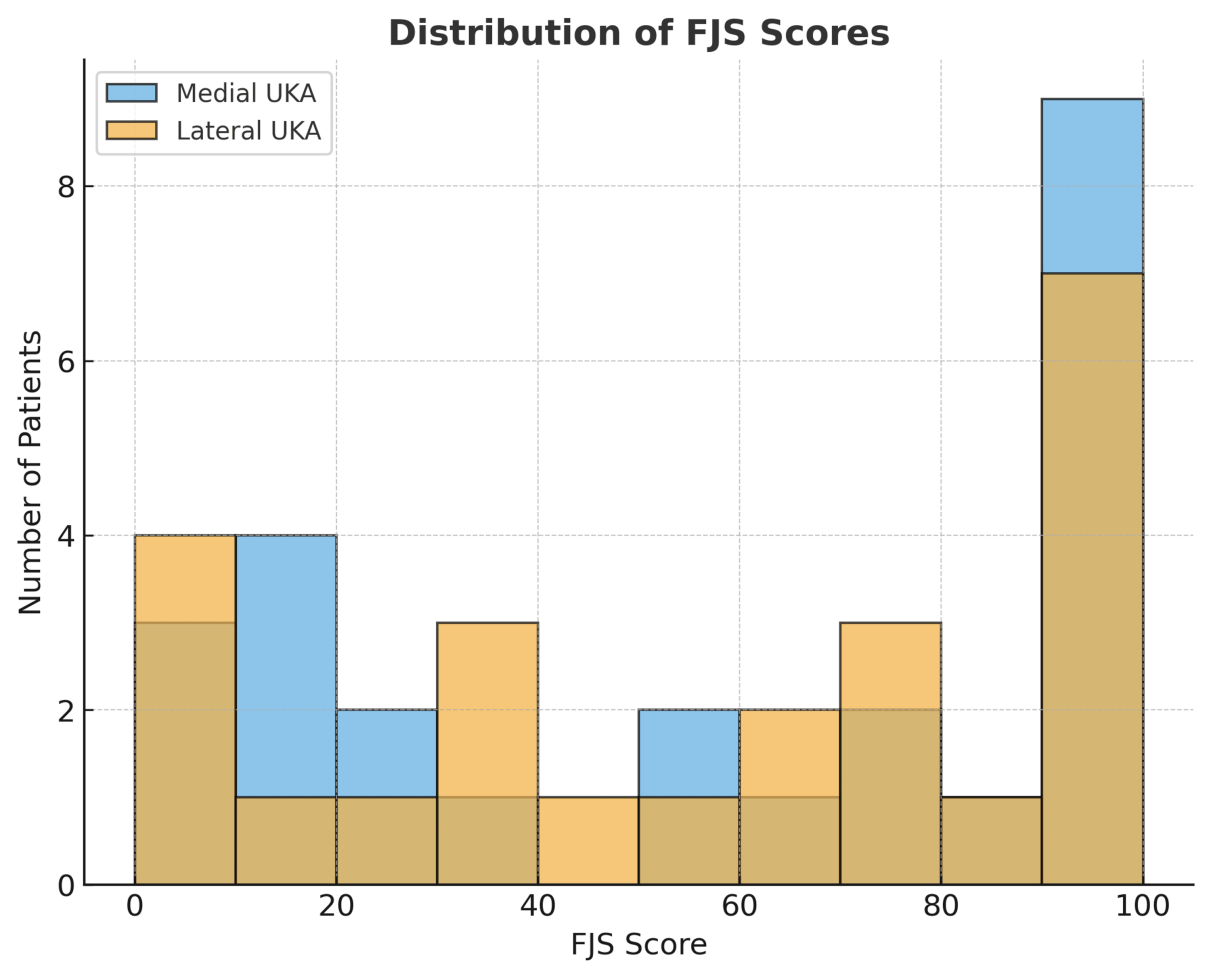
Histogram comparing medial and lateral UKA distribution of FJS-12 scores. UKA, unicompartmental knee arthroplasty; FJS, Forgotten Joint Score

**Figure 2:**
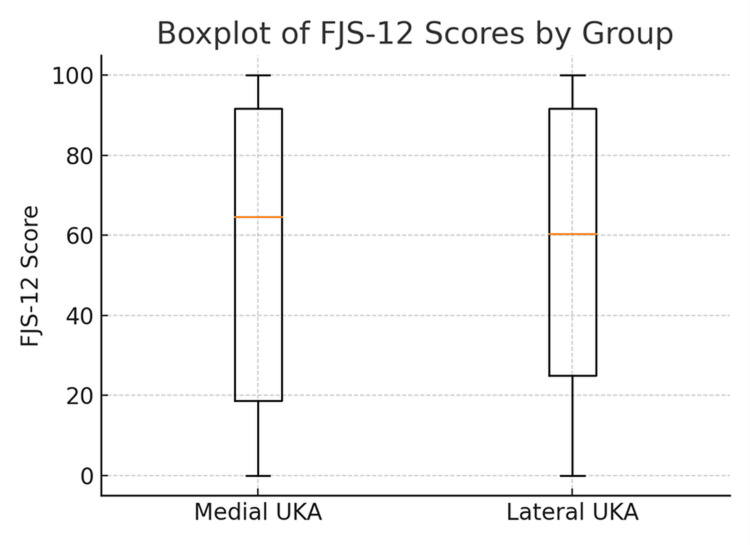
Boxplots comparing medial with lateral UKA distribution of normalized FJS-12 scores. UKA, unicompartmental knee arthroplasty; FJS-12, Forgotten Joint Score-12

At the latest follow-up, patient-reported outcome measures, as assessed by the Forgotten Joint Score-12 (FJS-12), were comparable between groups. All lateral UKA patients operated on by a single surgeon were contactable via telephone, and therefore, there were no missing lateral UKA numbers from our data. The mean normalized FJS-12 score was 56.8 (SD: 37.7) for medial UKA and 57.4 (SD: 36.0) for lateral UKA (p = 0.86), corresponding to a negligible effect size (Cohen’s d = 0.05). The item-by-item analysis of the FJS-12 questionnaire using the independent t-test p-value and Cohen’s d revealed no statistically significant differences in any of the 12 activity-specific domains, as shown in Table 2. The distribution of scores (illustrated by the histogram in Figure 1 and boxplots in Figure 2) showed substantial overlap between groups, indicating similar levels of joint “forgettability” in daily function. There were no records of implant revision in either of the two groups.

**Table 2:** Individual FJS-12 item analysis breakdown with the associated t-test statistic generating corresponding p-values. FJS-12: Forgotten Joint Score-12

FJS-12-assessed component	T-statistic	Cohen’s d	P-value
Walking on level ground	-0.29	-0.08	0.775
Walking on uneven ground	-0.29	-0.08	0.775
Standing from a low chair	1.32	0.37	0.197
Walking several blocks	-0.20	-0.06	0.842
Engaging in sports/recreation	0.22	0.06	0.830
Shopping	-0.64	-0.18	0.525
Washing yourself	0.46	0.13	0.651
Doing housework	-0.12	-0.03	0.908
Getting in/out of a car	0.23	0.07	0.817
Lying in bed	0.14	0.04	0.891
Climbing stairs	-0.65	-0.18	0.523
Standing for long periods	-0.80	-0.23	0.433

## Discussion

Our case-matched analysis compared medial with lateral UKA with respect to patient-reported outcomes. Medial UKA is more commonly performed owing to the higher incidence of medial compartment osteoarthritis, which is frequently associated with varus malalignment in the majority of the population. Lateral UKA, often associated with valgus malalignment and post-traumatic sequelae, is less commonly performed, and therefore, overall operative numbers in a given unit are often low [12]. This is reflected in the relative paucity of literature focused on lateral UKA in general, which in turn would limit the statistical power of such comparative studies [13]. A case-matched study provides a means for comparative analysis while minimizing variability with regard to patient demography and fundamental technical differences between the two procedures. Patient demographics, as well as follow-ups, were matched between the two groups to reduce potential bias. Additionally, in our methodology, the original operating team was not involved in the data collection to minimize interviewer bias. To the best of our knowledge, this is one of the few case-matched analyses for medial versus lateral UKA.

There are biomechanic and kinematic differences between the medial and lateral compartments of the knee, which demand distinct surgical modifications. The lateral compartment inherently has more femoral rollback and requires more complex soft tissue balance. Also, surgical exposure and ligament preservation are more challenging. Due to this increased rollback, mobile-bearing lateral UKAs have been reported to have higher rates of dislocation [14]. On the other hand, the medial compartment has more predictable joint biomechanics but requires careful attention to prevent varus overcorrection and to avoid increased forces across the lateral compartment. In our subset of cases, all of them were performed by a single surgeon using a medial parapatellar approach, reducing the bias associated with surgical exposure and dissection. Having no revision cases in our study prevented any significant statistical analysis and estimation of a hazard ratio, underlining the durability of both procedures. However, the literature does mention diverse patterns of failure for each. While medial UKA has a higher tendency to fail due to aseptic loosening, bearing dislocation especially for mobile-bearing prosthesis, and the progression of lateral compartment osteoarthritis, lateral UKA is more susceptible to poly wear and bearing dislocation due to increased rollback [13]. Among our cohort of patients, no distinct or compartment-specific group of failures was noted. This fortifies the significance of observing diligent technique in both procedures.

Our chosen patient-reported outcome measure (PROM) was the Forgotten Joint Score (FJS), which has, in previous studies, demonstrated good test-retest reliability and validity, placing it among other trusted markers such as the Oxford Knee Score (OKS) [15]. Our findings conclude comparable results between medial and lateral UKR in terms of FJS. The mean scores for medial and lateral UKA FJS outcomes were 56.8 and 57.4, respectively. The t-test p-value was 0.86, indicating no significant difference in PROMs between the groups. The histogram and boxplots included in our results (Figures 1, 2) show diagrammatically the similarity in the distribution of FJS scores between medial and lateral UKA. The FJS allows the researcher to measure a patient’s “joint awareness,” which is a good indicator of the true integration of an implant. This characteristic is overlooked by other validated PROMs such as the OKS or the Western Ontario and McMaster Universities Osteoarthritis Index (WOMAC). The scoring has a high internal consistency in prior studies [16]. The comparable outcomes in our study may reflect effective implant design, surgical technique, and appropriate patient selection.

We can compare our results in the context of similar studies in the literature. In a larger two-center retrospective cohort study conducted by Moye et al., two groups of 124 cases of medial and lateral UKA were analyzed for reoperation rates, functional performance, and patient-reported outcomes. These groups were matched by age, sex, body mass index, and year of procedure. When the Knee Injury and Osteoarthritis Outcome Scores (KOOS) were compared, there were equivalence outcomes in the groups [17]. However, the follow-up period was two years in comparison to our longer mid-term follow-up duration. In another two-year follow-up clinical trial using fixed-bearing implants, 119 medial UKA and 84 lateral UKA OKS scores showed similar outcomes between the two groups; however, this was not a case-matched study [10]. To our knowledge, there are no direct comparison studies using the FJS-12 as the primary PROM score comparing medial with lateral UKA.

There have been previous systematic reviews and meta-analyses examining outcomes between medial and lateral UKA. A meta-analysis by Bai et al. recently looked at 15 cohort studies comprising 2592 medial UKA and 614 lateral UKA and found no statistically significant differences between medial and lateral UKA in several domains [18]. These included functional scores, postoperative pain, complication rate, and implant survival [18]. However, most of the studies lacked long-term follow-up. Han et al. [19] undertook a meta-analysis of eight studies comprising 33999 medial UKAs and 2853 lateral UKAs and evaluated similar outcomes to Bai et al. [18]. They too found no significant differences between pain scores, implant longevity, and functional outcomes. There was heterogeneity in study designs, and a lack of long-term follow-up of >10 years was reported [19]. In both above analyses, there was a sample imbalance, with medial UKAs as expected by far outnumbering lateral UKA by operative volume. This may limit statistical power for lateral UKAs. In addition, high heterogeneity in study designs and outcome measures must be considered, as well as the limited availability of long-term data. Placing our study in the context of these larger combined analyses shows comparable conclusions and strengthens the notion that implant integration and functional outcomes remain similar between medial and lateral UKA.

Traditionally, there has been hesitancy among surgeons for lateral UKA. Our results unsubstantiated this notion and provided patient selection, and surgical techniques are optimized; it supports the larger adoption of lateral UKA in selected cases. However, surgeons should be aware of the steeper learning curve and the requirement of accurate soft tissue balancing. Other variables, such as the choice of implant design (fixed or mobile bearing), should be in accordance with the surgeon’s experience and familiarity.

The postoperative experience for a patient undergoing UKA is not unidimensional; it does not solely focus on the “forgettability” of an artificial joint. Pain, stiffness, and locking, to name but a few symptoms, are not evaluated in the FJS but can be captured in other PROMs, which is a limitation in this study. Despite this, FJS is a validated tool that does capture whether a patient is aware of the artificial joint during an array of activities of daily living. Another limitation of our study was the relatively modest sample sizes for a case-matched study. This restricts the statistical power for identifying infrequent incidents and minor differences. A single-surgeon design does amplify internal validity but does curb applicability to surgical practice in wider terms. Our mid-term follow-ups make it difficult to draw conclusions about long-term implant survival when failures associated with wear may turn up.

## Conclusions

In this novel case-matched analysis, both medial and lateral UKA cases demonstrated similar functional outcomes using the FJS-12, a validated PROM tool. The equivalent results of patient satisfaction between the groups in both the total score and individual activities indicate similar joint integration of medial and lateral UKA across a range of daily functions. These give support to the fact that lateral UKA is as safe and effective as the much more commonly performed medial UKA. Given the low operative volume of lateral UKA, our study adds to the growing evidence on patient-reported outcomes evaluating its use.
